# An Al-based system for fully automated knee alignment assessment in standard AP knee radiographs⋆

**DOI:** 10.1016/j.knee.2025.02.013

**Published:** 2025-03-03

**Authors:** Dominic Cullen, Peter Thompson, David Johnson, Claudia Lindner

**Affiliations:** a Division of informatics, Imaging and Data Sciences, School of Health Sciences, The University of Manchester, United Kingdom; b Northern Care Alliance NHS Foundation Trust, Salford, United Kingdom; c Department of Trauma and Orthopaedics, Stockport NHS Foundation Trust, Stepping Hill Hospital, Stockport, United Kingdom; d School of Health and Society, University of Salford, United Kingdom; e School of Biological Sciences, The University of Manchester, United Kingdom

**Keywords:** Clinical decision support system, Total knee arthroplasty, Knee osteoarthritis, Varus/valgus, Machine learning

## Abstract

**Background:**

Accurate assessment of knee alignment in pre- and post-operative radiographs is crucial for knee arthroplasty planning and evaluation. Current methods rely on manual alignment assessment, which is time-consuming and error-prone. This study proposes a machine learning-based approach to fully automatically measure anatomical varus/valgus alignment in standard anteroposterior (AP) knee radiographs.

**Methods:**

We collected a training dataset of 566 pre-operative and 457 one-year post-operative AP knee radiographs from total knee arthroplasty patients, along with a separate test set of 376 patients. The distal femur and proximal tibia/fibula were manually outlined using points to capture the knee joint. The outlines were used to develop an automatic system to locate the points. The anatomical femorotibial angle was calculated using the points, with varus/valgus defined as negative/positive deviations from zero. Fifty test images were clinically measured on two occasions by an orthopaedic surgeon. Agreement between points-based manual, automatic, and clinical measurements was assessed using intra-class correlation coefficient (ICC), mean absolute difference (MAD) and Bland-Altman analysis.

**Results:**

The agreement between automatic and manual measurements was excellent pre-/post-operatively with ICC 0.98/0.96 and MAD 0.8°/0.7°. The agreement between automatic and clinical measurements was excellent pre-operatively (ICC: 0.97; MAD: 1.2°) but lacked performance post-operatively (ICC: 0.78; MAD: 1.5°). The clinical intra-observer agreement was excellent pre-/post-operatively with ICC 0.99/0.95 and MAD 0.9°/0.8°.

**Conclusion:**

The developed system demonstrates high reliability in automatically measuring varus/valgus alignment pre- and post-operatively, and shows excellent agreement with clinical measurements pre-operatively. It provides a promising approach for automating the measurement of anatomical alignment.

## 1 Introduction

The prevalence of knee osteoarthritis (OA) in the UK is increasing. Currently, an estimated 5.4 million people in the UK are affected by the condition with projections suggesting this will increase further [1,2]. The most common reason for knee. arthroplasty is severe, end-stage OA. When all non-invasive measures have been exhausted and a patient is at end-stage knee OA, a collaborative decision is made between clinician and patient to perform more invasive treatments. One of the options is total knee arthroplasty (TKA) with over 75,000 primary TKAs conducted annually in the UK [3].

Alignment of the lower extremity has significant implications for both the progression of OA and outcomes of TKA. Excessive deviation from a neutral alignment at the knee joint, either laterally or medially, is classified as varus or valgus defor-mity/malalignment. Both varus and valgus deformities have been shown to be risk factors for OA progression in the knee [4]. Additionally, adequate post-operative alignment is considered one of the major criteria for primary TKA success. Suboptimal prosthesis placement with alignment in either varus or valgus has been linked with component loosening and instability, with an acceptable range being quoted between 2.4° and 7.2° of valgus [5,6].

Post-operatively, malalignment can lead to excessive mechanical and shear forces on the bearing surfaces and implant-to-bone interfaces. This malalignment jeopardises implant longevity by causing accelerated polyethylene wear, aseptic loosening of components, or even catastrophic failure. As a result, TKA requires meticulous attention to achieve optimal alignment during both pre-operative planning and intra-operative procedures to ensure proper restoration [7].

To assess knee alignment, radiographic imaging is required. Long-leg radiographs are used to measure the mechanical femorotibial angle (mFTA). However, long-leg radiographs are not always undertaken in clinical practice, and standard anteroposterior (AP) knee radiographs are often the main imaging modality. As a result, in standard AP knee radiographs where mFTA cannot be observed, the anatomical femorotibial angle (aFTA) is used as an alternative measurement. In a healthy population, aFTA is typically about 5-7° of valgus, and the amount of deformity is determined by the difference from that range. An aFTA greater than 7° indicates valgus deformities, while an aFTA lower than 0° indicates varus deformities [8,9].

Standard AP knee radiographs offer the potential to reduce costs and improve efficiency. In contrast, long-leg radiographs carry implications such as increased radiation to the pelvic organs and the need for specialised radiography equipment with attendant costs [10]. To make the appropriate measurements both pre- and post-operatively, clinicians currently use a variety of digital tools to accurately assess the degree of knee varus/valgus on radiographic images. This is often time-consuming and prone to reliability errors. In some cases, for less experienced surgeons, errors have occurred in template sizing vs actual sizing that have led to surgical complications [11].

Artificial intelligence (AI), particularly machine learning, has gained significant interest in orthopaedics recently, offering numerous opportunities for clinicians and patients. In this study, we aimed to explore the feasibility of automating knee alignment measurements in standard AP knee radiographs. Specifically, our objective was to develop and validate a machine learning-based system capable of automatically measuring varus/valgus alignment in pre- and post-operative standard AP knee radiographs.

## 2 Methods

### 2.1 Data

We collected a training dataset of 566 pre-operative and 457 one-year post-operative standard AP weight-bearing knee radiographs. Further, we have collected a separate distinct test dataset with both pre-operative and one-year post-operative AP weight-bearing knee radiographs for 376 patients. For ease of analysis and comparison, all right knee radiographs have been horizontally flipped to appear as showing a left knee. All radiographs were of patients who underwent TKA and did not have revision surgery before three years post-operatively. Some of the patients had sustained tibia/fibula fractures both prior to or after their TKA. The radiographs of these patients were included in the dataset to ensure that we were observing a complete population of patients, rather than a selected subset. The data was retrospectively collected from Stockport NHS Foundation Trust (approved by the Health Research Authority, IRAS 244130). All data were anonymised with any patient-identifiable information removed, and no patient-informed consent was required.

We used the training dataset to develop a machine learning-based automatic measurement system that can locate point positions and derive automatic measurements.

### 2.2 Automatic point placements

The automatic measurement system is based on our in-house software tool BoneFinder® (https://www.bone-finder.cum), which uses a Random Forest regression-voting Constrained Local Models (RFRV-CLM) points detection model [12]. This is a supervised machine learning-based approach that enables point positions to be detected in images with no human input. The technology can be run on any computer without the requirement for specific technical equipment, enhancing the translatability of such a system.

**Figure 1:**
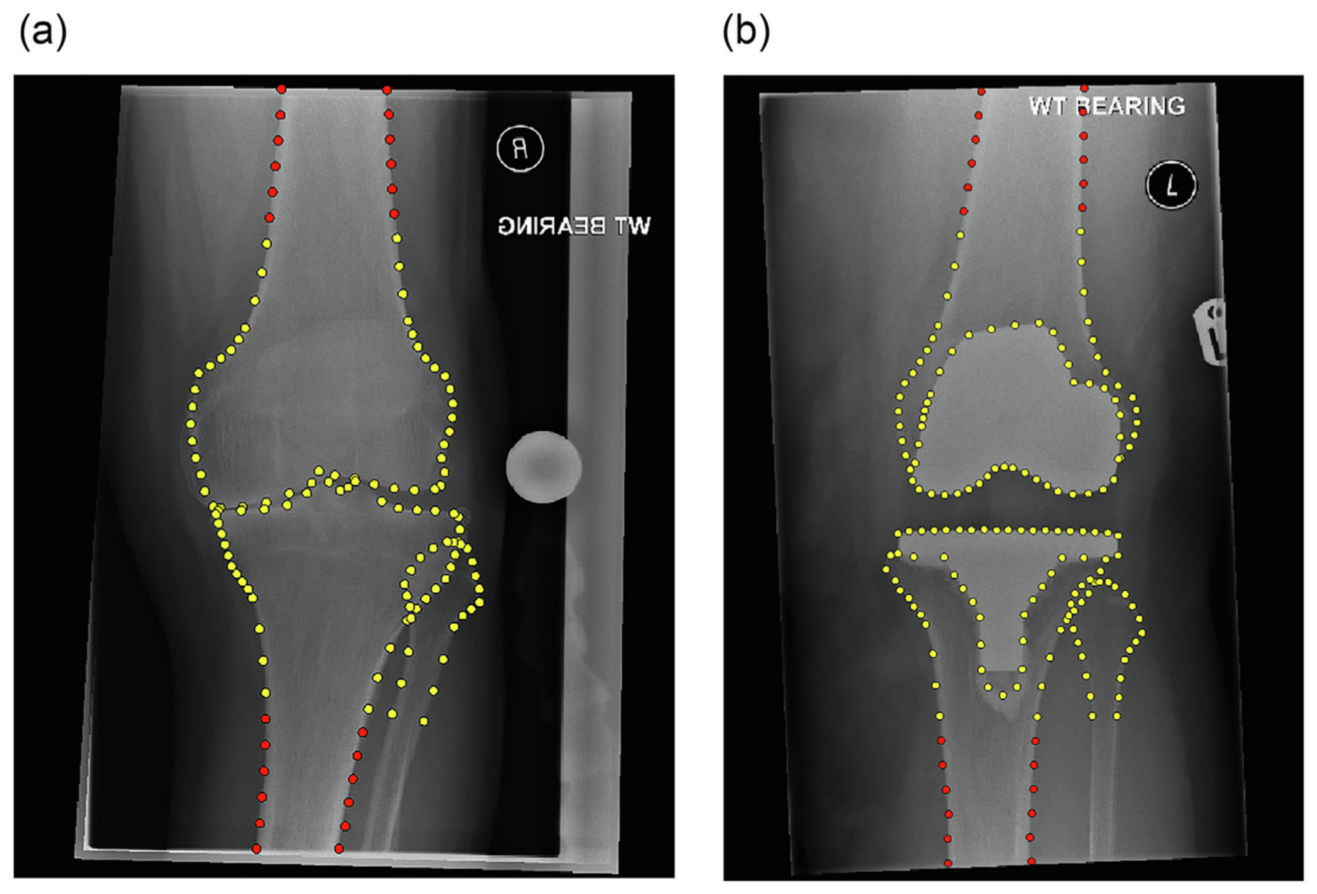
Annotation examples for the original/extended points sets (manually placed): (a) pre-operatively with 110/134 points, and (b) post-operatively with 157/181 points. The 24 extended points are highlighted in red. All radiographs have been cropped for better visualisation. Best viewed in colour. (For interpretation of the references to colour in this figure legend, the reader is referred to the web version of this article.)

Full details on how to train such a points detection model are given in [12,13], here we describe our experimental set-up. We trained four models, two for the pre-operative images and two for the post-operative images; based on an original and an extended points set. The original/extended pre- and post-operative models were trained to automatically detect 110/134 and 157/181 points, respectively (see. Figure 1). The points outlined the distal femur and the proximal tibia/fibula to capture the knee joint (including implants for the post-operative images). This included point positions used to permit calculation of aFTA.

To provide the ground truth for training and assessing the pre- and post-operative original/extended models, we manually annotated all 942 pre-operative and 833 post-operative training and test images with all 110 + 24 and 157 + 24 points, respectively. The original models did not include the extended 24 points along the femoral and tibial shafts (see Figure 1).

The pre- and post-operative point detection models were created using the respective manual annotations (i.e. point placements) of the training datasets. Each of the pre- and post-operative point detection models was then applied to the pre- and post-operative test dataset, respectively, to obtain automatic point positions for the test datasets.

### 2.3 Varus-valgus alignment measurement

We assessed varus/valgus alignment in pre-operative and post-operative standard AP knee radiographs by measuring aFTA. We defined varus/valgus as negative/positive deviations from zero, where the usual alignment is between +5° and +7°.

**Figure 2:**
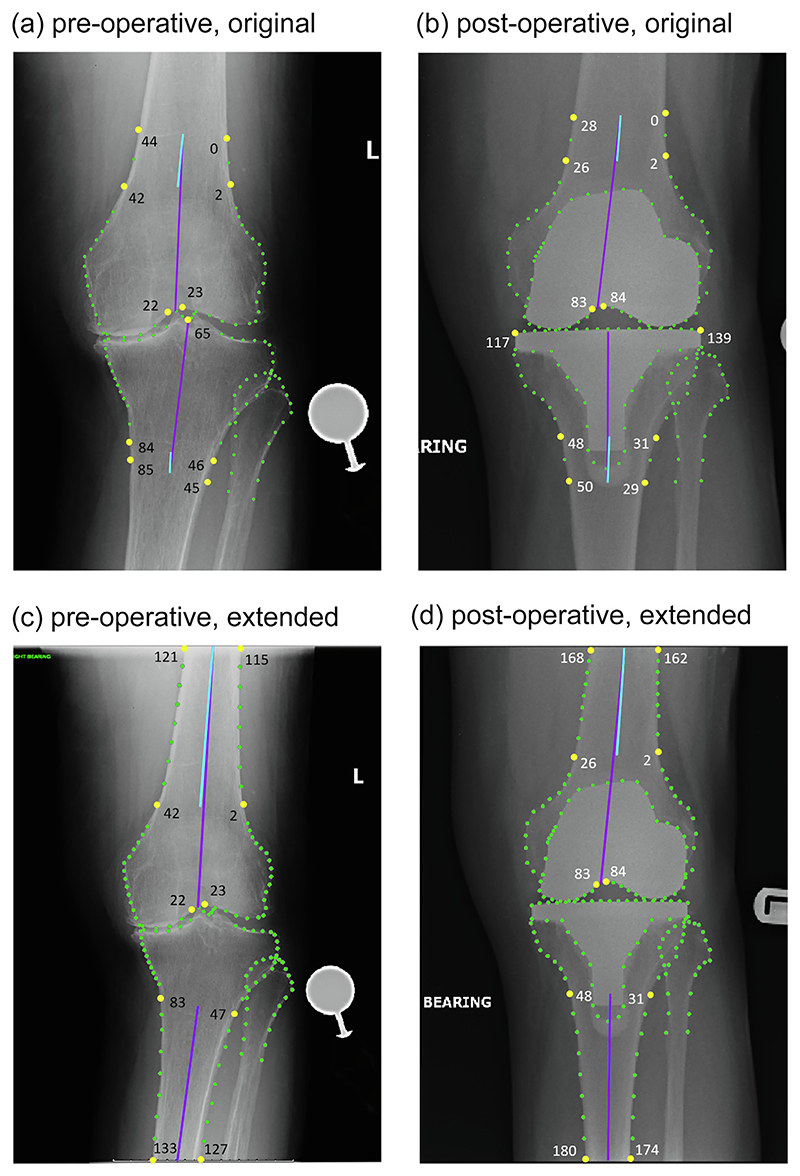
A diagram demonstrating how aFTA was calculated for the four different points sets, with blue indicating the shaft points only definition and purple the one also including the femoral notch or tibial spines. Blue and purple lines may be overlapping. For the measurements using the extended points sets, the blue and purple lines for the tibial shaft are the same and only the purple line can be seen. All radiographs have been cropped for better visualisation. Best viewed in colour. aFTA was defined as follows: **(a)** measurement *Pre-fem-tib-shafts* (blue) fits a centre line to the femur by connecting two shaft centre points (mid-points of points 44,0 and 42,2) and the tibia (mid-points of points 84,46 and 85,45), *measurement Pre-fem-notch-tib-spines* (purple) fits a line to the femur by connecting a shaft centre point (mid-point of points 44,0) to a femoral notch point (mid-point of points 22,23) and the tibia by connecting a shaft centre point (mid-point of points 85,45) with the tibial spine groove (point 65): **(b)** measurement *Post-fem-tib-shafts* (blue) fits a centre line to the femur by connecting two shaft centre points (mid-points of points 28,0 and 26,2) and the tibia (mid-points of points 48,31 and 50,29), measurement *Post-fem-notch-tib-spines* (purple) fits a line to the femur by connecting a shaft centre point (mid-point of points 28,0) to a femoral notch point (mid-point of points 83,84) and the tibia by connecting a shaft centre point (mid-point of points 50,29) with a tibial plateau centre point (mid-point of 117,139); (**c**) measurement *PreX-fem-tib-shafts* (blue) fits a centre line to the femur by connecting two shaft centre points (mid-points of points 121,115 and 42,2) and the tibia (mid-points of points 83,47 and 133,127), measurement *PreX-fem-notch-tib-shafts* (purple) fits a line to the femur by connecting a shaft centre point (mid-point of points 121,115) to a femoral notch point (mid-point of points 22,23) and the tibia by connecting two shaft centre points (midpoints of points 83,47 and 133,127); and (**d**) measurement *PostX-fem-tib-shafts* (blue) fits a centre line to the femur by connecting two shaft centre points (mid-points of points 168,162 and 26,2) and the tibia (mid-points of points 48,31 and 180,174), *measurement* PostX-fem-notch-tib-shafts (purple) fits a line to the femur by connecting a shaft centre point (mid-point of points 168,162) to a femoral notch point (mid-point of points 83,84) and the tibia by connecting two shaft centre points (mid-points of points 48,31 and 180,174). (For interpretation of the references to colour in this figure legend, the reader is referred to the web version of this article.)

We obtained three different types of aFTA measurements:

- **Manual measurements:** We calculated aFTA based on a subset of the *manually* annotated pre-/post-operative point positions as defined in Figure 2. This was applied to all images of the pre-/post-operative test datasets.
- **Automatic measurements:** We calculated aFTA based on a subset of the *automatically* located pre-/post-operative point positions as defined in Figure 2. This was applied to all images of the pre-/post-operative test datasets.
- **Clinical measurements:** For a random subset of 50 matched pre-/post-operative images from the test datasets, we had aFTA measured in the clinical setting using a PACS-integrated measurement facility by an orthopaedic surgeon (DJ). All images were measured twice, 7ȓ10 days apart and blinded to the first set of measurements. The mean of each pair of measurements provided the ground truth for the clinical measurements.

For the points-based manual and automatic measurements, we explored two different ways of calculating the measurements from the point positions: based on the tibial and femoral shaft points only as well as also including information about the femoral notch and tibial spines (see Figure 2).

We used several methods to assess the agreement between the automatic and manual as well as between the automatic and clinical aTFA measurements for the test dataset:

- The intra-class correlation coefficient (ICC) between the manual/clinical and automatic measurements; ICC scores are usually interpreted as poor below 0.5, moderate below 0.75, good below 0.9 and excellent from 0.9 [14].
- The mean absolute deviation (MAD), defined as the average of the absolute difference between the manual/clinical measurement and the automatic measurement for each image.
- A Bland-Altman analysis, which provides a graphical and statistical assessment of the agreement between the manual/-clinical and automatic measurements, highlighting any potential bias and the extent of agreement [15].

Further, we assessed the intra-observer variability for the clinical measurements in the same way.

## 3 Results

The pre- and post-operative point detection models located all knee joints in the test dataset correctly and no image was excluded for the results shown below.

### 3.1 Automatic vs manual varus-valgus alignment measurement

The results of comparing the points-based automatic and manual measurements are shown in Table 1. These show that the measurements based on only femoral and tibial shaft points perform significantly worse for the original models, as would be expected from the limited cover of the shafts in these models (see Figure 1).

The best pre-operative results with an excellent ICC of 0.98 were achieved for the extended model when not only relying on the shaft points but also including information about the positioning of the femoral notch in the aFTA definition (i.e. measurement PreX-fem-notch-tib-shafts). Post-operatively, the best agreement with an excellent ICC of 0.96 was achieved for the original model when including both the femoral notch and the tibial plateau centre point into the aFTA definition (i.e. measurement Post-fem-notch-tib-spines).

**Figure 3:**
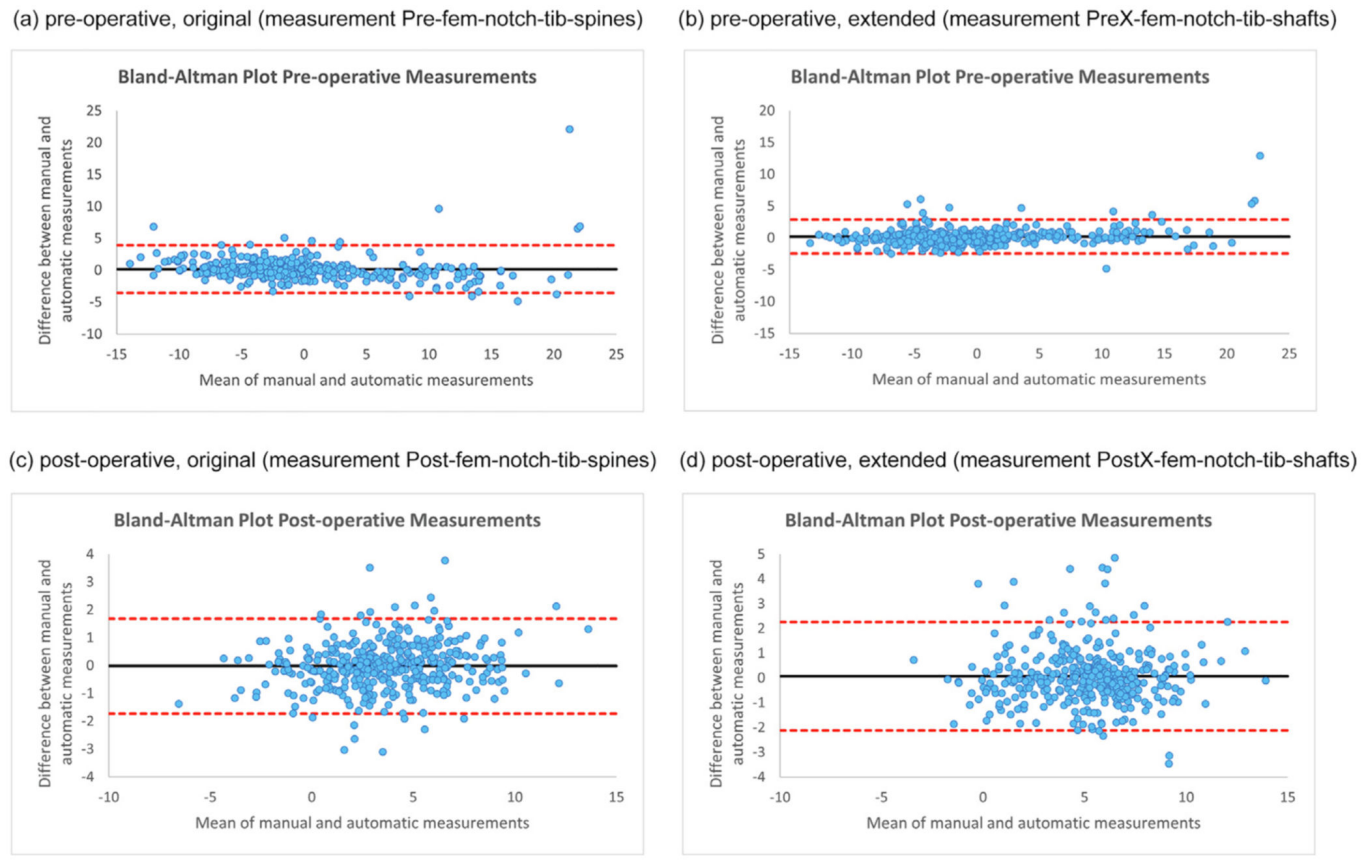
Bland-Altman plots tor the best-pertorming measurement systems when comparing automatic with manual measurements (showing results for all 376 knee radiographs of the independent test dataset). (For interpretation of the references to colour in this figure legend, the reader is referred to the web version of this article.)

Figure 3 shows the Bland-Altman plots for the best pre-operative/post-operative, original/extended models as defined by ICC in Table 1. The plots visualise that none of the models is biased as well as that the limits of agreements for the post-operative models are lower.

**Figure 4:**
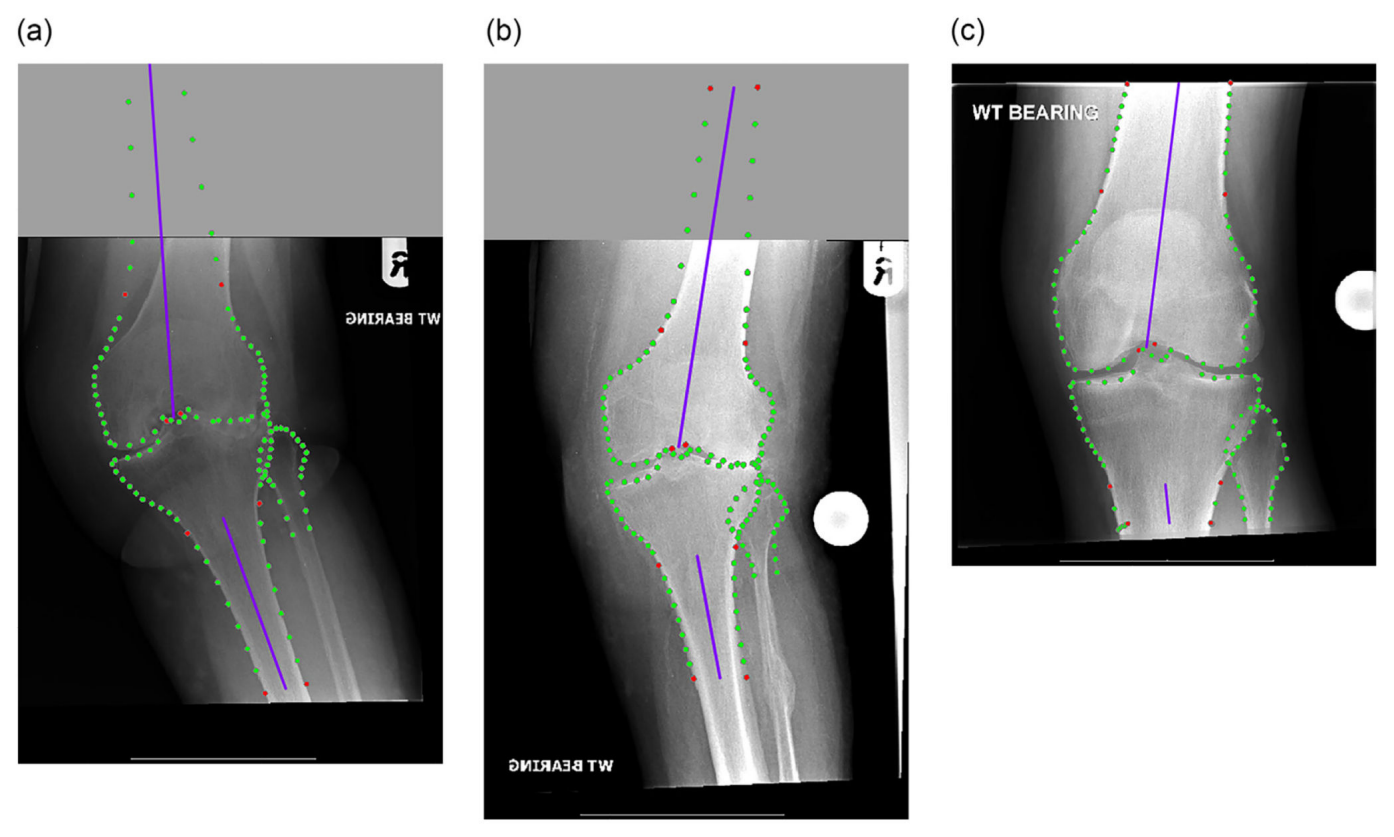
Poor-performing outliers of results obtained with best model for pre-operative measurement PreX-fem-notch-tib-shafts when *compared to manual annotations:* (a) absolute difference: 14.3° (worst case result); (b) absolute difference: 7.9; and (c) absolute difference: 7.0°. Best viewed in colour. (For interpretation of the references to colour in this figure legend, the reader is referred to the web version of this article.)

**Figure 5:**
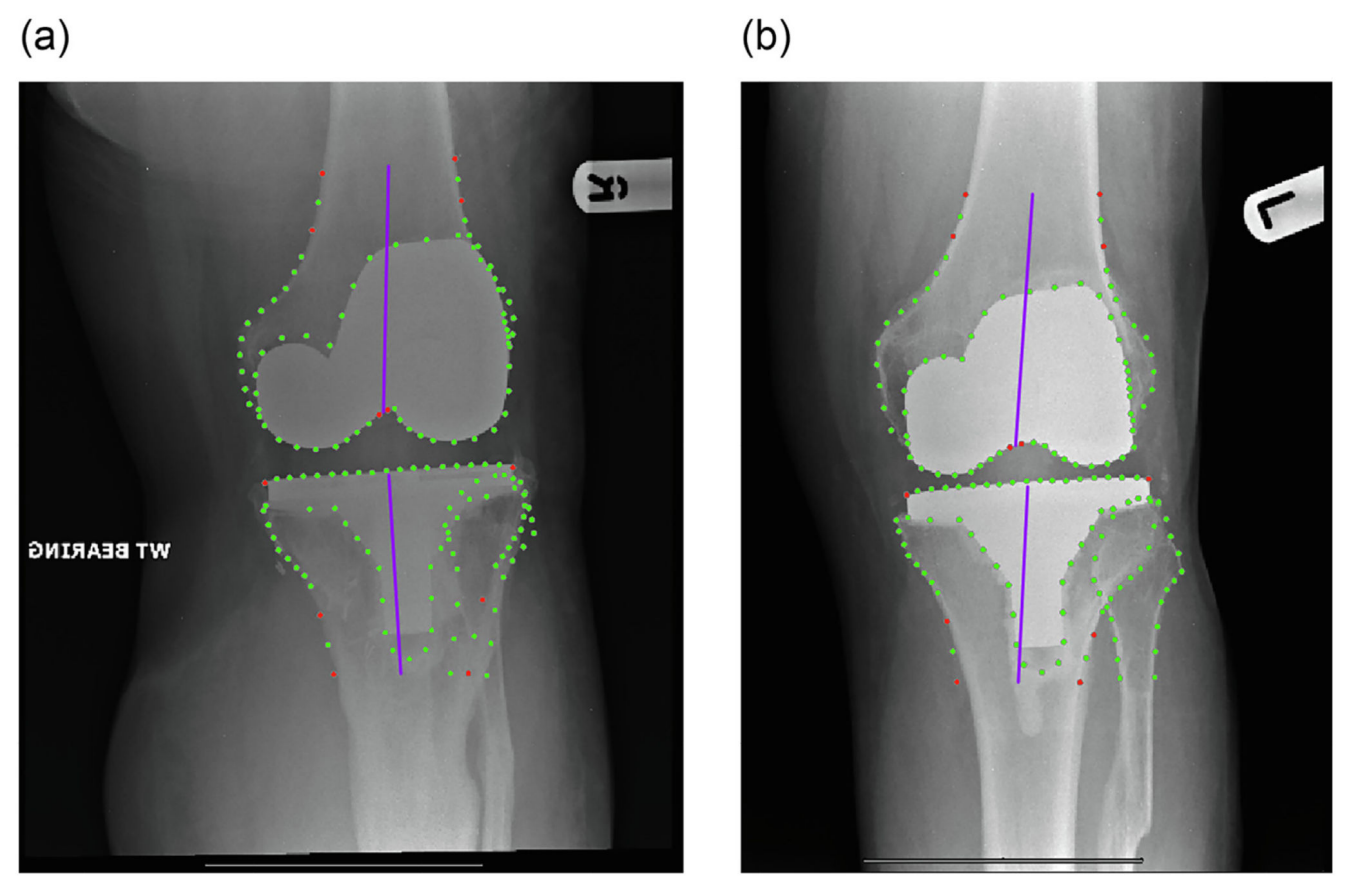
Worst cases of results obtained with best model for post-operative measurement Post-fem-notch-tib-spines when *compared to manual annotations:* (a) absolute difference: 3.8° (worst case result); and (b) absolute difference: 3.5°. Best viewed in colour. (For interpretation of the references to colour in this figure legend, the reader is referred to the web version of this article.)

The Bland-Altman plot for the best pre-operative system (extended model with measurement PreX-fem-notch-tib-shafts) when compared to manual annotations (Figure 3b) shows one extreme outlier with an absolute difference of 14.3°, the worst-performing image. The search result and line fitting for this image is shown in Figure 4a. This demonstrates that this is a radiograph with a high valgus angle and a very short femoral shaft. Two further poor-performing outliers of the same system are given in Figure 4b and Figure 4c, and also show a rather short femoral or tibial shaft, respectively. Figure 4b also demonstrates a united fracture of the proximal fibula. No such apparent outlier is visible for the best post-operative system (original model with measurement Post-fem-notch-tib-spines) when compared to manual annotations (Figure 3c). However, the worst cases are visualised in Figure 5, with Figure 5a incompletely showing a healed upper third tibia and fibula fracture, and Figure 5b showing a proximal third fibula fracture.

For the best post-operative system (original model with measurement Post-fem-notch-tib-spines), 58.5% and 54.0% for the points-based automatic and manual measurements, respectively, were within the acceptable alignment range of between 2.4° and 7.2° of valgus [6].

### 3.2 Automatic vs clinical varus-valgus alignment measurement

The results of comparing the points-based automatic and the clinical measurements for a random subset of 50 knees from the test dataset are shown in Table 2. The results show excellent agreement for the pre-operative images but only moderate agreement for the post-operative ones.

The best pre-operative results with an excellent ICC of 0.97 were achieved for the extended model when not only relying on the shaft points but also including information about the positioning of the femoral notch in the aFTA definition (i.e. measurement PreX-fem-notch-tib-shafts). For this setting, the agreement between the automatic and clinical measurements was comparably good to the agreement between the automatic and manual measurements. The post-operative systems performed poor to moderate when compared to the clinical measurements (with the best results obtained when approximating the tibial axis using only tibial shaft points). This is in contrast to the excellent agreement between the automatic and the manual measurements. Of note is that the results in Table 1 and Table 2 are not directly comparable as the latter is based on a much smaller subset of the data.

Figure 3 shows the Bland-Altman plots for the best pre-operative/post-operative, original/extended models as defined by ICC in Table 2.

**Figure 6:**
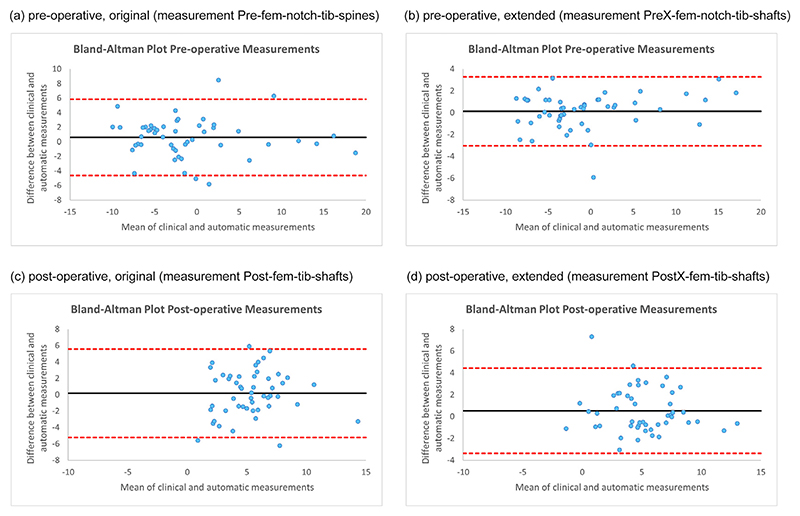
Bland-Altman plots for the best-performing measurement systems when comparing automatic with clinical measurements (showing results tor **a** random subset of 50 knee radiographs from the independent test dataset). (For interpretation of the references to colour in this figure legend, the reader is referred to the web version of this article.)

**Fig. 7:**
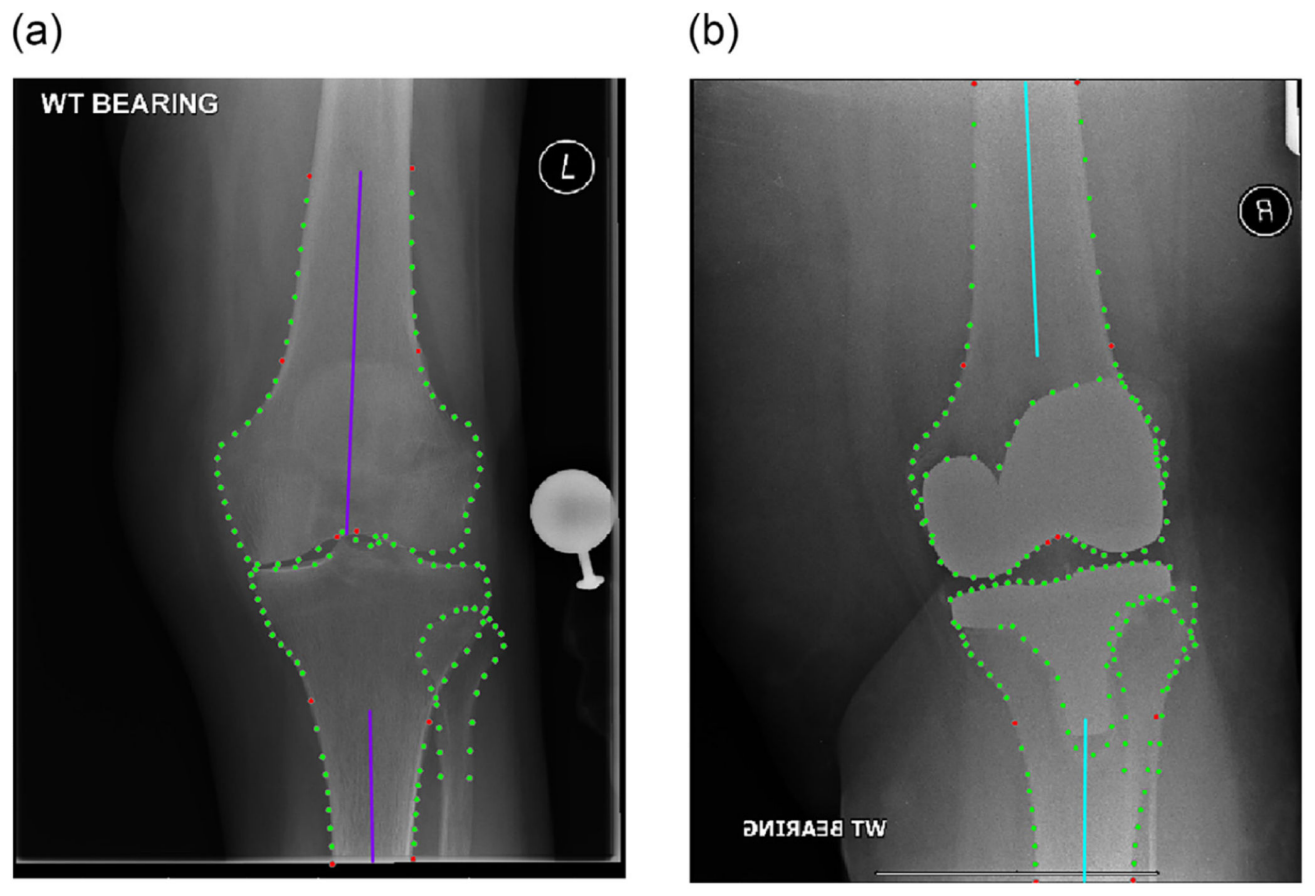
Worst cases of results obtained with best pre- and post-operative models when *compared to clinical measurements* for: (a) pre-operative measurement PreX-fem-notch-tib-shafts, absolute difference: 5.9°; and (b) post-operative measurement PostX-fem-tib-shafts, absolute difference: 7.3°. Best viewed in colour. (For interpretation of the references to colour in this figure legend, the reader is referred to the web version of this article.)

The Bland-Altman plot for the best pre-operative system (extended model with measurement PreX-fern-notch-tib-shafts) when compared to clinical measurements (Figure 6b) shows one poor-performing outlier with an absolute difference of 5.9°. The search result and line fitting for this image is shown in Figure 7a, showing a good quality well-aligned extended radio-graph. Similarly, the Bland-Altman plot for the best post-operative system (extended model with measurement PostX-fem-tib-shafts) when compared to clinical measurements (Figure 6d) shows one poor-performing outlier with an absolute difference of 7.3°. The search result and line fitting for this image is shown in Figure 7b, showing a rotated radiograph.

For the best post-operative system (extended model with measurement PostX-fem-tib-shafts), 56% and 54.0% for the points-based automatic and clinical measurements, respectively, were within the acceptable alignment range of between 2.4° and 7.2° of valgus [6].

### 3.3 Intra-observer clinical varus-valgus alignment measurement

The results of the intra-observer variability analysis of the clinical measurements are given in Table 3. The results show excellent agreement with an MAD of 0.9° for the pre-operative images and 0.8° for the post-operative images. The results suggest that the clinical measurements are slightly more consistent for the post-operative images. For the pre-operative images, the results are comparable to the best-performing automatic measurement system which uses the extended points and defines aFTA including information about the positioning of the femoral notch in the aFTA definition (i.e. measurement PreX-fem-notch-tib-shafts).

In our experiments, conducted on a standard laptop, the automatic extended pre- and post-operative models took on average 1ȓ2 min to search a single image. The post-operative extended model took the longest, while the original models were faster. This is because no run-time optimisation was implemented and each point was searched for one after the other (i.e. models with more points took longer). Significant speed-up could be obtained by taking advantage of parallel processing (i.e. let the model search for multiple points at the same time). Using the point positions to calculate one or multiple measurements takes the system less than one second per image.

## 4 Discussion

### 4.1 Key findings

This study focused on the development and validation of a machine learning-based fully automatic system to calculate aFTA in pre-/post-operative standard AP knee radiographs. The system demonstrated strong reliability in measuring varus/-valgus deformities in pre-operative radiographs. These findings suggest that machine learning-based tools can play a pivotal role in enhancing the consistency and efficiency of radiographic assessments in knee arthroplasty.

For the best pre-operative measurement system, the agreement between the automatic and manual measurements as well as between the automatic and clinical measurements was comparably excellent. This suggests that automatically measuring aFTA cannot only be done reliably but also that the proposed points-based definition agrees with the way the measurement is taken in a clinical environment.

With regards to the automatic vs manual performance on post-operative images, the limits of agreements are lower for the post-operative models, suggesting that the automated models are able to more consistently generate the post-operative measurements. This is likely because there is significantly less variation in knee shape in the post-operative images compared to the often severely pathologic pre-operative knees. The latter is also indicated by the significantly tighter mean value range for the post-operative results compared to the pre-operative ones. However, the post-operative, models only performed poor to moderate when compared to the clinical measurements. This conflicting performance of the post-operative automatic system in comparison to the manual vs clinical measurements indicates that while the post-operative measurements can be reliably generated automatically, the used points-based definitions of aFTA for the post-operative images do not agree with the way that the measurement is taken in a clinical environment.

We explored different ways on how to algorithmically define aFTA based on point positions outlining key structures of the femur and tibia. For the original models, we found that shaft-based approaches did not produce reliable results as the parts of the femoral and tibial shafts that were covered did not provide sufficient information to represent the femoral and tibial axes accurately. Extending the coverage of the femoral and tibial shafts lead to significant improvements in being able to automatically estimate the femoral and tibial axes. For pre-operative images, best performance was achieved by defining the femoral axis using the extended shaft information in combination with information about the position of the femoral notch and the tibial axis was best approximated using tibial shaft points only (see measurement PreX-fem-notch-tib-shafts in Figure 2). For the post-operative images, while we are able to reliably generate automated measurements, the proposed algorithmic points-based definitions of aFTA do not agree with the way aFTA is measured in clinical practice.

The small differences in the proportion of cases within the acceptable alignment range between points-based automatic and manual measurements as well as between points-based automatic and clinical measurements, indicate that the automatic system performs comparably to manual methods and aligns reasonably well with defined clinical thresholds. Cases falling outside the acceptable range may reflect true alignment deviations in patients or may result from the limitations discussed below.

### 4.2 Limitations

This study focusses only on a single measurement of knee alignment, aFTA, and as such can be a proof-of-concept for automating knee alignment measurements in AP, and potentially lateral, knee radiographs. Further, the comparison to the clinical measurements is limited in that we only have clinical measurements from a single observer, for a comparatively small subset of 50 independent test images. No information on inter-observer variation is available. However, research on manually measuring aTFA on knee radiographs suggests that the inter-observer variation maybe in the range of the automatic results presented in this study, with Ilahi et al. showing a measurement difference of within 3.1° for 95% of the images and a maximum difference of 6° [16]. In addition, in our study we have not investigated the impact of image quality on performance, for example, with regards to leg rotation or the visible length of the femur and tibia. The two aspects may affect the reliability and repeatability of knee alignment measurements from standard radiographs. Jamali et al. have shown that rotation of as small as 3° leads to a statistically significant difference in alignment measurements such as aFTA [17], Sgroi et al. found that at least 20 cm of each the femoral and tibial bones would need to be shown in an AP knee radiograph to ensure reliable and repeatable alignment measurements [18]. The length of the visible bones in our dataset has not been quantified. However, qualitatively we have observed that in most images at least one of the bones is of significantly less length. Examples of this are shown in Figures 4, 5 and 7. For the pre-operative images, the largest disagreement was found for a radiograph with a high valgus angle and a very short femoral shaft (see Figure 4a). In this case, the pre-operative extended points detection model likely failed to outline the femur as images with such a high valgus angle are not well represented in the data that was used to train the system. In addition, the very short femoral segment does not provide much guidance to the system to predict the actual alignment of the femoral shaft. The short length of the visible femur and tibia in AP radiographs may explain why a large proportion of the post-operative alignment measurements fall outside the acceptable range in our study.

Further to the limitations of short and/or rotated radiographs, the imaged tissues may also affect measurements. Of our outlier images a number were identified as having sustained previous fractures. This may be associated with deformity at the level of the joint in both coronal and sagittal plane, where measurements may be more difficult to assess clinically. In addition, the presence of a fracture may affect the automated system’s ability to detect an accurate outline of the bone and any malunion may also affect measurements. For the post-operative radiographs, the orientation of the implants may affect the normal patterns used to assess images either automatically or clinically.

Finally, even with perfect radiographs and relatively normal anatomy, a clinical observer may measure an image differently but consistently for reasons which are not clear. This would merit further exploration to ensure that outlier status can be attributed to either the clinician or automated method as the latter may potentially affect the information provided to any decision-making tool. Indeed, it may be that it would be preferable to take the system’s measurement in preference, to the clinician’s measurement as, aside from being more consistent, it may correlate more strongly with clinical outcomes, which we propose to explore in future studies.

### 4.3 Comparison with previous studies

There is a growing body of research that proposes and evaluates Al-based systems to measure knee alignment in pre- and post-operative long-leg radiographs [19–23]. However, we have deliberately focussed on standard AP knee radiographs which represents daily clinical practice in many healthcare settings. This imaging modality for planning and assessing knee arthroplasty has been the focus of only limited research to date. Wang et al. explored using an Al-based system to directly predict knee alignment measurements from PA radiographs, and achieved high accuracy (FTA mean absolute error: 0.8°) on pre-operative radiographs from the Osteoarthritis Initiative (OAI) database [24], Of note is that the OAI radiographs have been taken in a standardised way and do not reflect the variation encountered in clinical practice as is present in our data, including image rotation and reduced shaft length (as discussed above).

### 4.4 Future work

To improve the automatic measurement system and its assessment, we suggest the following next steps: (i) perform inter-observer analysis for clinically obtained measurements to put results of the automatic system into perspective with current clinical practice; (ii) expand the measurements set to include additional measurements of knee/implant alignment; (iii) perform analysis comparing standard AP knee radiographs with long leg radiographs; (iv) analyse radiographic bone texture to assess the quality of bone-cement-implant interfaces; and (v) identify the performance of the automatic system depending on image quality to define requirements. The latter may then lead to the development of a quality score that can indicate whether a radiograph is of sufficient quality for being measured automatically. Alternatively, the system could measure every image and provide a confidence score as to the accuracy of the measurement along with the measurement.

For the post-operative images, the high agreement between the manual and automatic points-based measurements but the reduced agreement between the points-based and clinical measurements indicates that we will need to better understand on how to algorithmically define aFTA in post-operative images.

The use of machine learning in the knee arthroplasty pathway offers numerous future opportunities. In the long-term, the overall goal is to extend the AI system to inform decision-making about potential outcome following arthroplasty, and to identify patients that need closer post-operative follow-up. We aim to achieve this by combining the outcome of the proposed measurement system with other clinical and patient-reported data to detect and predict failure, where failure may be defined as either poor patient-related outcomes or early implant failure, including signs of implant loosening or wear.

### 4.5 implications for practice

Digital innovation, particularly through AI tools, has the potential to transform the entire knee arthroplasty pathway. The integration of the Getting it Right First Time (GIRFT) pathway has taken the initial steps of how to standardise the knee arthroplasty pathway and the continuation of this approach will provide new data that can be interpreted for its efficacy [25]. There are already examples of how digital innovation may assist the GIRFT pathway [26]. We propose that a system such as the one alluded to in this work could be integrated in the same way.

From initial diagnosis to post-operative care, machine learning could streamline processes, reduce variability, and support decision-making. Pre-operatively, machine learning-based tools have the potential to assist in patient selection, predicting which individuals are most likely to benefit from arthroplasty based on a combination of clinical, imaging, and demographic data. Intra-operatively, machine learning-driven robotic assistance could ensure optimal implant positioning and alignment, reducing the likelihood of revision arthroplasty. Post-operativeiy, machine learning-based tools can potentially monitor recovery and identify patients at risk of complications, enabling timely interventions [27,28].

While the benefits of machine learning in the knee arthroplasty pathway are significant, there are also limitations and ethical considerations to address. The reliance on high-quality datasets for training machine learning-based systems may lead to biases if the data is not representative of diverse patient populations. Ensuring transparency in the development and validation of machine learning-based systems is crucial for fostering trust among clinicians and patients alike [29].

## 5 Conclusion

The integration of machine learning into the knee arthroplasty pathway offers promising opportunities to improve patient outcomes through enhanced precision, personalised treatment plans, and streamlined processes. The proposed fully automatic measurement system provides a promising proof-of-concept for automating anatomical knee alignment measurements in pre- and post-operative standard AP knee radiographs. The point-detection models to automatically outline the knee in pre- and post-operative AP knee radiographs are freely available to the research community via https://wwwbone-finder.com. This will not only enable replication of results but also allow further studies into identifying how to best define a points-based aFTA measurement for post-operative radiographs.

## Figures and Tables

**Table 1:** Summary of performance of automatic pre- and post-operative measurement systems when *compared to manual measurements* (as defined by manually placed point positions) for an independent test dataset of 376 standard knee radiographs. (ICC = inter-correlation coefficient, MAD = mean absolute difference, CI = confidence interval, SD = standard deviation, BA = Bland-Altman).

	ORIGINAL models		EXTENDED models	
	Pre-/Post-fem-tib-shafts	Pre-/Post-fem-notch-tib-spines	PreX-/PostX-fem-tib-shafts	PreX-/PostX-fem-notch-tib-shafts
	***Pre-operative***			
**ICC (95% CI)**	0.87 (0.85−0.89)	**0.96 (0.95**−**0.97)**	0.97 (0.96−0.97)	**0.98 (0.97**−**0.98)**
**MAD (SD)**	2.4° (±2.2°)	1.1° (±1.6°)	1.0° (±1.3°)	0.8° (±1.1°)
**BA bias (SD)**	0.0° (±3.2°)	0.2° (±1.9°)	0.2° (±1.6°)	0.2° (±1.4°)
**BA limits of agreement**	±6.3°	±3.7°	±3.2°	±2.7°
	***Post-operative***			
**ICC (95% CI)**	0.70 (0.65−0.74)	**0.96 (0.95**−**0.96)**	0.88 (0.86−0.90)	**0.92 (0.90**−**0.93)**
**MAD (SD)**	1.9° (±1.5°)	0.7° (±0.6°)	0.9° (±1.1°)	0.8° (±0.8°)
**BA bias (SD)**	−0.4° (±2.4°)	−0.0° (±0.9°)	0.2° (±1.4°)	0.1° (±1.1°)
**BA limits of agreement**	±4.7°	±1.7°	±2.7°	±2.2°

**Table 2:** Summary of performance of automatic pre- and post-operative measurement systems when *compared to clinical measurements* (taken in a clinical set-up) for a random subset of 50 standard knee radiographs from the independent test dataset. (ICC = inter-correlation coefficient, MAD = mean absolute difference, CI = confidence interval, SD = standard deviation, BA = Bland-Altman).

	ORIGINAL models		EXTENDED models	
	Pre-/Post-fem-tib-shafts	Pre-/Post-fem-notch-tib-spines	PreX-/PostX-fem-tib-shafts	PreX-/PostX-fem-notch-tib-shafts
	***Pre-operative***			
**ICC (95% CI)**	0.88 (0.81−0.92)	**0.92 (0.87**−**0.95)**	0.95 (0.91−0.97)	**0.97 (0.95**−**0.98)**
**MAD (SD)**	2.4° (±1.9°)	2.1° (±1.8°)	1.4° (±1.4°)	1.2° (±1.0°)
**BA bias (SD)**	−0.7° (±3.1°)	0.6° (±2.7°)	−0.8° (±1.8°)	0.1° (±1.6°)
**BA limits of agreement**	±6.0°	±5.2°	±3.6°	±3.2°
	***Post-operative***			
**ICC (95% CI)**	**0.54 (0.35**−**0.69)**	0.39 (0.15−0.58)	**0.78 (0.67**−**0.86)**	0.71 (0.56−0.81)
**MAD (SD)**	2.2° (±1.5°)	2.9° (±2.0°)	1.5° (±1.3°)	1.7° (±1.5°)
**BA bias (SD)**	0.2° (±2.7°)	1.7° (±3.1°)	0.5° (±2.0°)	0.8° (±2.2°)
**BA limits of agreement**	±5.4°	±6.1°	±3.9°	±4.2°

**Table 3:** Intra-observer agreement for clinical measurements based on two sets of clinical measurements for a random subset of 50 standard knee radiographs from the independent test dataset. (ICC = intercorrelation coefficient, MAD = mean absolute difference, Cl = confidence interval, SD = standard deviation, BA = Bland-Altman).

	*Pre-operative*
**ICC (95% CI)**	0.99 (0.98−0.99)
**MAD (SD)**	0.9° (±0.6°)
**BA bias (SD)**	−0.3° (±1.0°)
**BA limits of agreement**	±2.0°
	***Post-operative***
**ICC (95% CI)**	0.95 (0.92−0.97)
**MAD (SD)**	0.8° (±0.5°)
**BA bias (SD)**	−0.1° (±0.9°)
**BA limits of agreement**	±1.8°
